# Supplementary material for: A Global Screen for Assembly State Changes of the Mitotic Proteome by SEC-SWATH-MS
Source: Cell Syst. 2020 Feb 26;10(2):133–155.e6. doi: 10.1016/j.cels.2020.01.001 (PMC7042714; doi:10.1016/j.cels.2020.01.001)

P25106 | ACKR3_HUMAN | ACKR3 CMKOR1 CXCR7 GPR159 RDC1
Monomer MW [kDa]: 41.493 Monomer expected elution fraction: 48

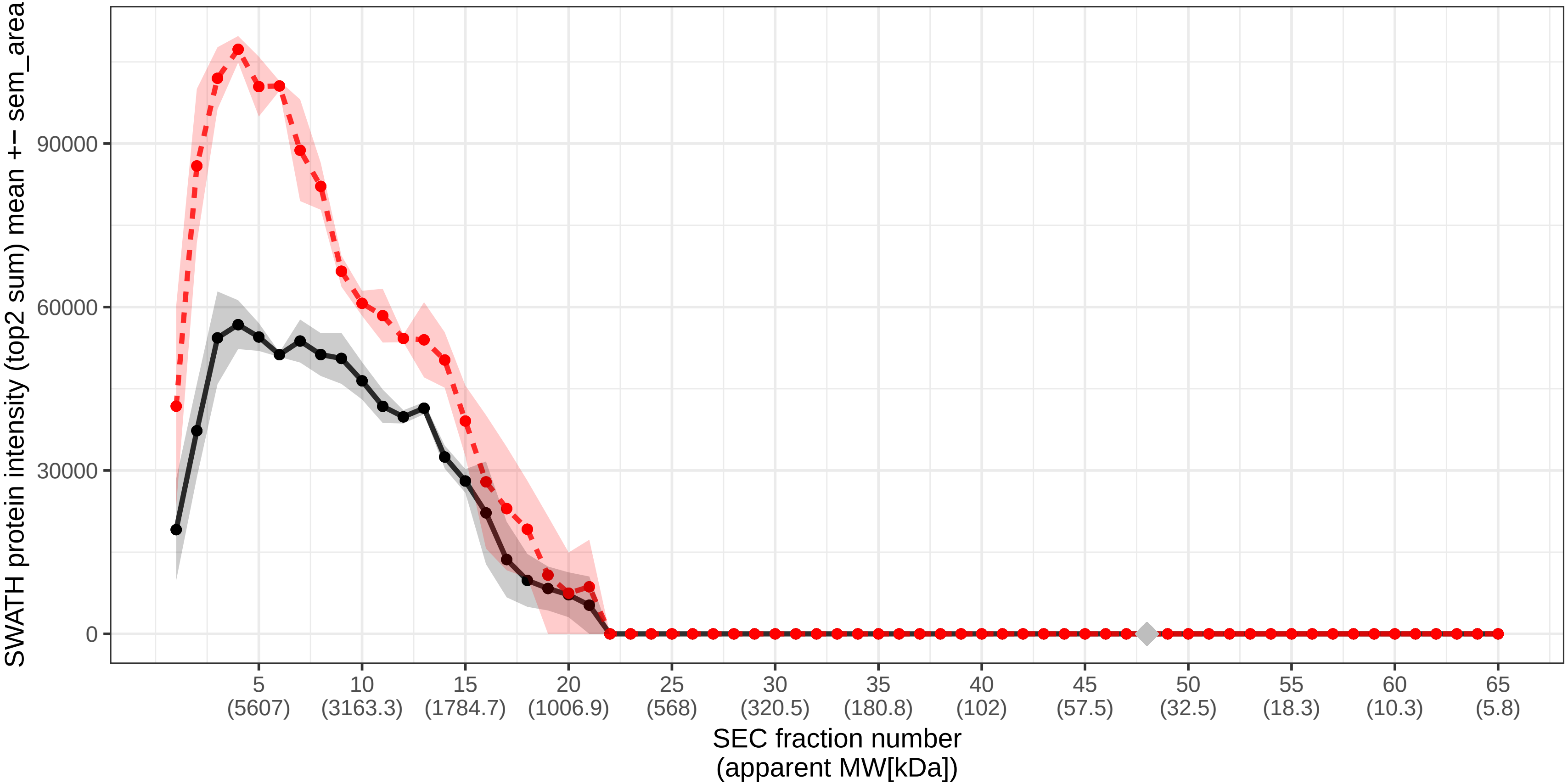

Supplement: Data S1. SEC-SWATH-MS Protein Chromatograms, Related to Figure 1 [file mmc6.zip › SECchrom_P25106_ACKR3_HUMAN_ACKR3_CMKOR1_CXCR7_GPR159_RDC1.pdf]
